# Cytogenetic and FISH analysis of 93 multiple myeloma Moroccan patients

**DOI:** 10.1002/mgg3.1363

**Published:** 2020-06-23

**Authors:** Hasna Hamdaoui, Oumaima Benlarroubia, Oum Kaltoum Ait Boujmia, Hossein Mossafa, Karim Ouldim, Aziza Belkhayat, Imane Smyej, Houda Benrahma, Hind Dehbi, Fatima Chegdani

**Affiliations:** ^1^ National Reference Laboratory Mohammed VI University of Health Sciences (UM6SS) Casablanca Morocco; ^2^ Laboratory of Health and Environment Faculty of Sciences Ain Chock University Hassan II Casablanca Morocco; ^3^ Laboratory of Cellular and Molecular Pathology Faculty of Medicine and Pharmacy of Casablanca University Hassan II Casablanca Morocco; ^4^ National Reference Laboratory Mohammed VI University of Health Sciences (UM6SS) Casablanca Morocco; ^5^ Faculty of Medicine and Pharmacy Medical Genetics and Oncogenetics Unit Sidi Mohamed Ben Abdellah University Fes Morocco; ^6^ Faculty of Medicine Mohammed VI University of Health Sciences (UM6SS) Casablanca Morocco

**Keywords:** conventional karyotype, cytogenetic, FISH, multiple myeloma, plasma cells sorted

## Abstract

**Background:**

Multiple myeloma (MM) is a disease characterized by heterogeneous clinical presentations as well as complex genetic and molecular abnormalities. In MM, cytogenetic analysis is a challenge because of the low proliferation of malignant plasma cells. Thus, interphase fluorescence in situ hybridization (FISH), performed on sorted plasma cells detected abnormalities independently of a proliferative and infiltrative index. The purpose of this study was to explore, for the first time, the cytogenetic and molecular genetics features in Moroccan patients with multiple myeloma referred exclusively to National Reference Laboratory and to determine their risk stratification based on these features.

**Methods:**

We performed cytogenetic analysis on 93 MM cases, all patients were subjected to FISH analysis, among which 45 patients have benefited from both FISH analysis and standard karyotype.

**Results:**

Karyotype was normal in 78% (35/45) while, it was complex with varied structural and numerical abnormalities in 22% (10/45) of all patients, among which Hyperdiploid karyotype was found in 9% (*n* = 4 cases) and nonhyperdiploid in 13% (*n* = 6 cases). The most common numerical abnormalities were gains of chromosomes 3, 5, 9, 15, and 19. Whole chromosome losses were also frequent, affecting chromosomes X, 3, 14, 16 and 22. FISH analysis detected abnormalities in 50% of cases. The translocation t(4;14) and dup (1q) were the most frequent types of anomalies (14% and 13% respectively), followed by (17p) deletion and 14q32/*IGH* translocations with an undetermined origin (12% each) then the (1p) deletion (4%). For the normal karyotypes, FISH revealed chromosome abnormalities in 46%.

**Conclusion:**

This study compares the results of cytogenetic analysis of chromosomal abnormalities in the Moroccan population with other countries. ½ patient showed at least one type of molecular genetic abnormalities. Therefore, the introducing of the cytogenetic analysis is obligatory in the diagnosis of multiple myeloma.

## Graphical abstract

It's the first study of its kind, which studies the cytogenetic profile of multiple myeloma on Moroccan patients. In order to determine the frequency of the most recurrent anomalies in our population and their risk stratification based on these features.

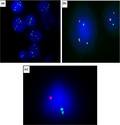

## 1 INTRODUCTION

Multiple myeloma (MM) is a clonal bone marrow disease characterized by the neoplastic transformation of differentiated B cells, which produce nonclonal immunoglobulin (Ig) or Ig fragments (Mprotein; Mohamed, Bentley, Bonnett, Zonder, & Al‐Katib, 2007; Sawyer, 2011). MM represents about 10% to 15% of all hematopoietic neoplasms, it is the second most frequent type of hematologic cancer with a frequency of 1% of all cancer cases, and the death from this type cancer represents 2% of all cancer deaths. The median age at diagnosis was 60 years old (Avet‐Loiseau et al., 2007; Hartmann et al., 2011; Kyle et al., 2003; Palumbo & Anderson, 2011). In some cases, an intermediate asymptomatic, more advanced premalignant stage named smoldering (or indolent) multiple myeloma (SMM) can be observed. It progresses to myeloma at a rate of 10% per year over the first 5 years following diagnosis, 3% per year over the following 5 years and 1.5% per year thereafter (Moreau et al., 2017). This disease is usually incurable, with a median survival rate of 3 to 4 years, and only 10% of patients survive more than 10 years (Hartmann et al., 2011; Mohamed et al., 2007).

Diagnosis of MM should be based on the following criteria: clonal bone marrow plasma cells ≥10% or biopsy‐proven bony or extramedullary plasmacytoma, and any one or more of the following myeloma defining events: Hypercalcaemia Renal insufficiency Anemia Bone lesions.

The criteria for diagnosis of MM were updated in 2014 by the International Myeloma Working Group (IMWG; Moreau et al., 2017; Rajkumar et al., 2014). The main revision was to add three very specific malignancy biomarkers: Clonal bone marrow plasma cell percentage ≥60%, involved/uninvolved serum free light chain ratio ≥100, and more than 1 focal lesions on MRI studies.

Treatment should be initiated in all patients with MM according to the updated definition proposed by the IMWG in 2014, by removing the need for documented end‐organ damage as a mandatory requirement for the definition of malignancy (Moreau et al., 2017; Rajkumar et al., 2014).

In MM, cytogenetic abnormalities affect clinical presentation, progression of smoldering multiple myeloma (SMM) to MM (Rajan & Rajkumar, 2015) and influence the diagnosis (Kumar et al., 2012; Neben et al., 2013; Rajkumar et al., 2013). It plays a major part in the risk stratification; into three groups: high risk, intermediate risk, and standard risk; due to prognostic impact of various cytogenetic abnormalities as well as to the association between emerging therapeutic approaches in MM (DaudignonQuilichini & Ameye, 2016).

However, the study of cytogenetic abnormalities by karyotyping is limited because of the low proliferative activity of the malignant plasma cells in vitro^4^. The abnormal karyotype is found in about 30%–50% of MM cases (Kishimoto, de Freitas, & Ratis, 2016; Mohamed et al., 2007). Molecular studies have demonstrated that chromosomal aberrations can be detected by interphase fluorescence in situ hybridization (iFISH) technique as the most useful cytogenetic tool for their investigations, in noncycling interphase cells.

iFISH testing does not depend on cell proliferation, it needs a presorting of CD138 positive plasma cells (Avet‐Loiseau et al., 2007; Daudignon et al., 2016) to detect abnormalities independently of proliferative index (Daudignon et al., 2016). Many previous studies have demonstrated that chromosomal aberrations in MM are complex, involving many chromosomes that are altered both numerically and structurally (Mohamed et al., 2007; Sawyer, 2011).

Therefore, the aim of the present study was to explore, for the first time, the cytogenetic and molecular genetics features in Moroccan patients with multiple myeloma (MM) and determine their risk stratification based on this features.

## 2 PATIENTS AND METHODS

### 2.1 Ethical compliance

The study was approved by the internal ethics committee of the Cheikh Khalifa International University Hospital and the Mohammed VI University of Health Sciences (UM6SS), Casablanca, Morocco.

### 2.2 Patients

Between May 2017 and June 2018, 93 heparinized bone marrow (BM) samples were collected from Moroccan patients diagnosed with multiple myeloma (MM) who presented Clonal bone marrow plasma cells >10% plus one or more of the CRAB features:hypercalcemia, renal insufficiency, anemia, bone lesions, and myeloma‐defining events. Ninety‐three BM samples were sent to national reference laboratory of Cheikh Khalifa Foundation, the Military hospital of Rabat, The Military hospital of Meknes, University hospital center of Fez and too many other laboratories in different regions of Morocco (Fez, Marrakech, Tangier…) for chromosomal analysis and Fluorescence in situ hybridization (FISH) analysis. Among the 93 patients, 45 have benefited from both FISH analysis and standard karyotype analysis while 48 patients have benefited only from FISH analysis.

### 2.3 Metaphase chromosomes/conventional cytogenetic

Cytogenetic studies were performed on 96‐hr bone marrow (BM) cultures. The culture medium used was RPMI 1,640, supplemented with 20% fetal bovine serum and 2% l‐glutamine (100×). After incubation, cultures were exposed to karyoMax colcemid solution (0.1 μg/ml) for 30 min followed by a hypotonic (0.075 mol/L) treatment. Washes in Carnoy's fixative (methanol–acetic acid, 3:1) were done by harvesting process by HANABI PII Metaphase Chromosome Harvester. R‐banding was performed by treating the slides in a sodium di‐hydrogen phosphate 1‐hydrate solution (NaH2PO4 H2O) warmer at 87.4°C for 12 min, and then stained with Giemsa solution. A minimum of 20 metaphases per case were analyzed. Karyotypes were described according to the International System for Human Cytogenetic Nomenclature ISCN (2016; Daudignon et al., 2016).

### 2.4 Magnetic cell sorting

Magnetic cell separation of PCs was performed using the Whole Blood CD138 MicroBeads, Whole Blood Column Kit, and the QuadroMACS Separation Unit (Miltenyi Biotec) according to the manufacturer's protocol. First, 1 ml of heparinized bone marrow specimen was passed through a 200‐μm preseparation filter (Miltenyi Biotec) to remove cell clumps and bone fractions. Next, 50 μl of MicroBeads were added to the cell suspension and incubated for 15 min at 4°C. The cell suspension was washed to remove the unbound antibodies and then transferred to the separation column. After removal from the magnetic field, the immunomagnetic‐labeled cells were eluted from the column.

### 2.5 Flow cytometry analysis of magnetic cell sorting enrichment

The MACS enrichment was verified by flow cytometry analysis. The unpurified bone marrow sample and enriched PC population were evaluated for their PC percentage. At least 50 μl of the magnetic cell sorting enrichment sample (MACS) were evaluated by flow cytometry. Anti‐CD138‐PO, anti‐CD38‐APC‐H7, and anti‐CD45‐PE‐Cy5 monoclonal antibodies were used to identify bone marrow PCs. Flow analysis was carried out in FACS CantoII equipment. Data analysis was performed using the BD FACS Diva software. PCs were identified by their high expression of CD38.

### 2.6 FISH procedure

#### 2.6.1 Harvest procedure

Three milliliters of hypotonic potassium chloride solution (0.075 M KC) were added to the MACS enrichment sample and incubated for 25 min at 37°C. And then 2 ml of fixative solution (Carnoy's solution: 3:1 methanol/acetic acid) was added and the tube was centrifuged at 1,500 rpm for 10 min. The supernatant was discarded; the pellet was re‐suspended in 4 ml of Carnoy's solution and centrifuged again at 1,500 rpm for 5 min. The latter procedure was repeated two more times and the resulting pellet was used in the FISH procedure.

#### 2.6.2 Interphase fluorescence in situ hybridization

Slides were pretreated according to the manufacture's protocol (Daudignon et al., 2016). The FISH probes used in this study included *IGH/FGFR3*(4p16/ 14q32; DC.DF)/vysis, *TP53/CEP 17*(17p11.1‐q11.1/ 17p13.1) FISH Probe, vysis and 1q21 *CKS1B*/ 1p32 *CDKN2C*FISH Probe, vysis. FISH analysis of probe hybridization was performed with a 100× objective fluorescence microscope (Genasis, ADS Biotec) with single and triple emission filters. iFISH results were described according to the standards of the International System for Human Cytogenetic Nomenclature (ISCN) 2016 (Daudignon et al., 2016).

### 2.7 Statistical analysis

Statistical analysis was performed with the statistical package for Social Sciences SPSS version 20 (SPSS Inc.). The values *p* < .05 are considered to be significant.

## 3 RESULTS

### 3.1 Age and sex distribution

**Figure 1:**
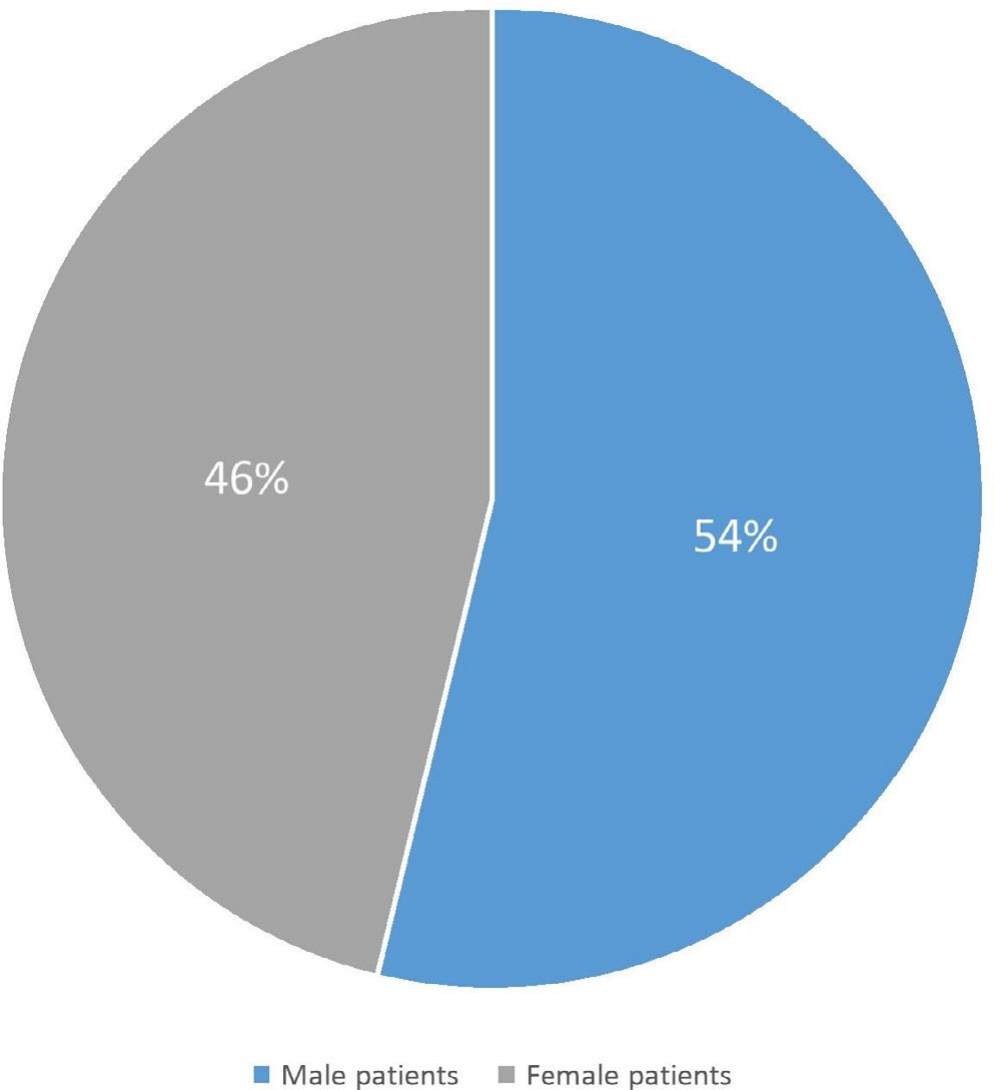
Gender distribution in the multiple myeloma cases

**Figure 2:**
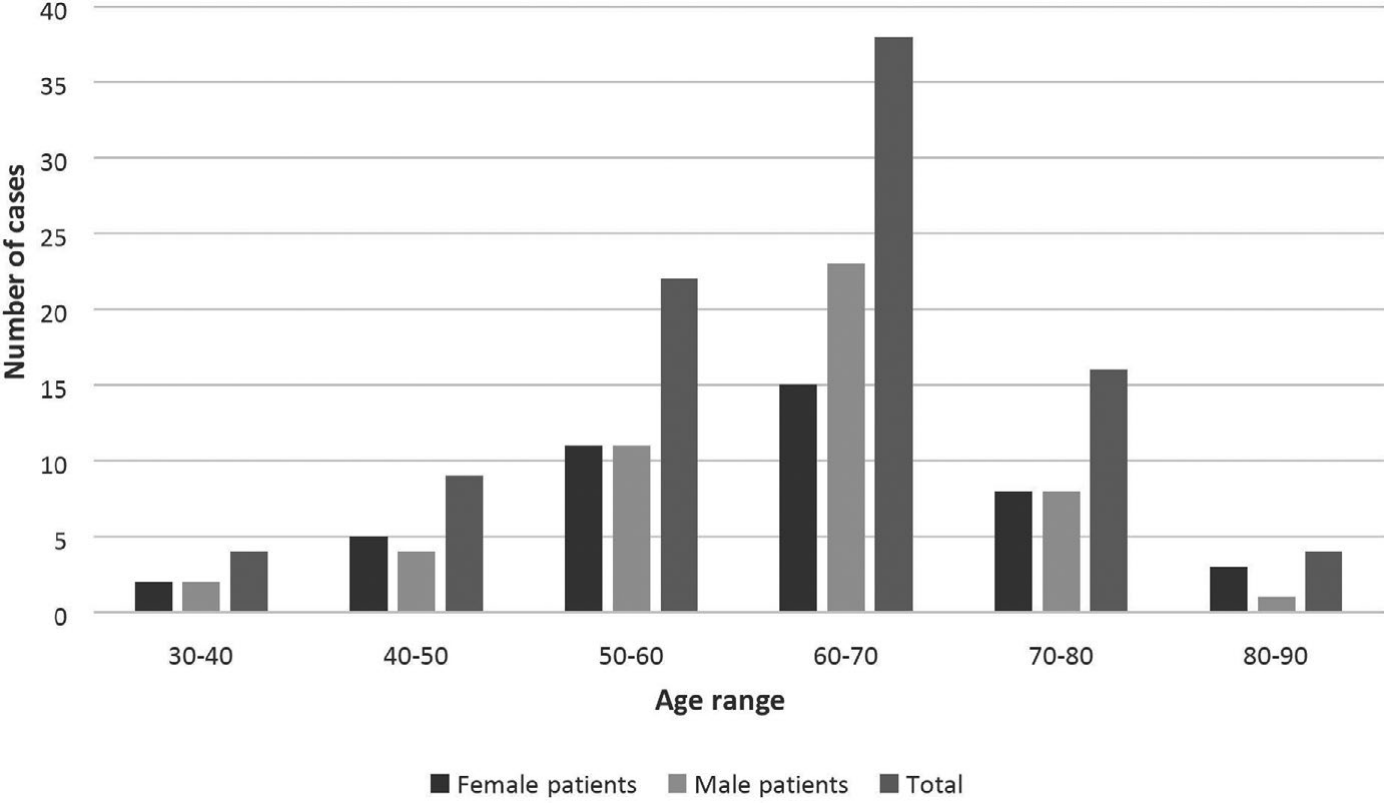
Age and gender distribution in the multiple myeloma studied cases

In the present study, the median age of all patients was 61.88 years old at diagnosis. MM affected men (54%) more than women (46%) with a sex ratio of 1.16 Figure 1. As shown in Table 1, patients were aged between 30 to 90 years old, 41% were in the age group of 60 to 70 years with male predominance Figure 2.

**Table 1:** Age and gender distribution of the 93 studies in multiple myeloma cases

Age range	Male patients	Female patients	Percentage
30–40	2	2	4%
40–50	4	5	10%
50–60	11	11	24%
60–70	23	15	41%
70–80	8	8	17%
80–90	1	3	4%
Total	49	44	100%

### 3.2 Cytogenetic analysis results

For the karyotype analysis, 78% (35/45) of patients had a normal karyotype while 22% (10/45) of patients had an abnormal karyotype with various or complex chromosomal abnormalities. The results of karyotyping are shown in Table 2.

**Table 2:** Detailed FISH Results of multiple myeloma patients with Complex karyotype

Age/gender	Karyotype result	% of plasma cells	% of % CD138 + cells	Used probes	FISH result
	Hyperdiploidy				
61M	51,XY,+der(1)del(p34p13),+der(2)t(2;?)(p25;?),+5,+9,der(12)t(12;?)(p13;?), del(16)(p12),+18,der(20)t(20;?)(p13;?)[3]/46,XY[22]	29%	93%	IGH/FGFR3: t(4 ;14) Del TP53 en 17p13 Del 1p36 Ampli 1q25	Absence Absence Presence Presence
63M	49‐50,XY,der(3)t(3;?)(P22;?),del(3)(P21),+del(6)(q16q23),+7,‐8,+del(9)(q12q22),‐11,add(13)(p11),+add(15)(P11),+21[cp4]/ 46,XY[14]	24.2%	95%	IGH/FGFR3: t(4 ;14) Del TP53 en 17p13 Del 1p36 Ampli 1q25	Presence Absence Absence Absence
41M	52‐53,XY,del(1)(p31),+3,+5,t(8;22)(q24,q12),+9,+12, +15,+18,+19,add(20)(q13),‐21,+mar[cp20]	not done	not done	IGH/FGFR3: t(4 ;14) Del TP53 en 17p13 Del 1p36 Ampli 1q25	Absence Absence Presence Absence
50F	50‐51,X,‐X,+3,der(4)t(4;?)(p13;?),+5,‐6,+7x2,der(8)t(8;?)(p12;?),+9x2,del(9) (q12q31),‐13,+15,del(17)(q22),+19[cp15]/46,XX[3]	49.1%	99.2%	IGH/FGFR3: t(4 ;14) Del TP53 en 17p13 Del 1p36 Ampli 1q25	IgHrearrange Absence Absence Absence
	Nonhyperdiploidy				
68F	82‐87,XX,‐4,‐5,+del(6)(q13q23),+del(7)(q22q34),del(8)(q12q23),‐10,t(11;14) (q13;q32),der(13)t(13;?)(p10;?),‐13,‐15,‐17x2,‐18,‐20,der(21;?) (p12;?),‐22x2,+mar1x3,+mar2x2,+mar3x2,+mar4[cp6]/46,XX[9]	15%	82%	IGH/FGFR3: t(4 ;14) Del TP53 en 17p13 Del1p36 Ampli 1q25	Presence Presence Absence Presence
68F	44,XX,der(1)t(1;21)(q11;q11),del(2)(p11.2p25),t(3;14)(p21;q32),del(4)(q13),t(8;?;1)(q24.2;?;p32),‐14,‐22[19]/46,XX[9]	22%	92,00%	IGH/FGFR3: t(4 ;14) Del TP53 en 17p13 Del1p36 Ampli 1q25	IgH rearrange Presence Absence Absence
45F	43,X,‐X,‐6,del(10)(23),‐13,der(14)t(14;?)(q32;?),‐16x2,‐19,+mar1,+mar2,+mar3[1]/ 46,XX[15]	17,50%	23%	IGH/FGFR3: t(4 ;14) Del TP53 en 17p13 Del 1p36 Ampli 1q25	IgH rearrange Absence Absence Absence
53F	45‐46,X,‐X,der(1)del(1p34),+der(1)del(1q21),t(11;14) (q23;q32),‐15,‐16,‐17,+mar1,+mar2[cp7]/46,XX[13]	3%	65%	IGH/FGFR3: t(4 ;14) Del TP53 en 17p13 Del 1p36 Ampli 1q25	IgH rearrange, Presence Absence Absence
48M	46,XY,del(6)(q21),del(9)(p13),t(11;14)(q13;q32),del(13)(q22),‐22,+mar[cp14]/ 46,XY[1]	Not done	80	IGH/FGFR3: t(4 ;14) Del TP53 en 17p13 Del 1p36 Ampli 1q25	IgH rearrange Absence Absence Absence
62M	46,XY,dup(1)(q21q32),+del(3)(q21),der(6)add(6)(p22)add(6)(q25),der(7)t(7;?) (p21;?),der(9)t(9;?)(p13;?),t(11;14)(q13;32),‐13[cp13]/46,XY[2]	7%	50%	IGH/FGFR3: t(4 ;14) Del TP53 en 17p13 Del 1p36 Ampli 1q25	IgH rearrange, Absence Absence Presence

The complex karyotype group (with numerical and structural chromosomal abnormalities) consisted of four cases of hyperdiploidy (47 < *n* < 53 chromosomes) representing 40% of the abnormal karyotypes, 9% of the total karyotypes, and six cases of nonhyperdiploidy (Hypodiploid, pseudodiploid, and near‐tetraploid karyotypes) with a frequency of 60% of the abnormal karyotypes and 13% of the total karyotypes.

#### 3.2.1 Hyperdiploidy versus Nonhyperdiploidy

##### Hyperdiploidy with 47–53 chromosomes (*n* = 4)

Four patients displayed hyperdiploid karyotypes (involving gains of odd‐numbered, 3 (*n* = 2), 5 (*n* = 3), 9 (*n* = 4), 15 (*n* = 3), 19 (*n* = 2), with an incidence of 9% of the total karyotypes and 40% of abnormal karyotypes. It was usually conjuncted with heterogeneous additional structural chromosomal aberrations such as 1p deletion in two cases, the translocation affecting band 8q24 (the site of *CMYC* oncogene (OMIM190080) contained the Burkitt's‐type): t(8;22)(q24,q11) translocation involving *IGL/MYC*(OMIM 147220)/(OMIM 190080) in one case.

**Figure 3:**
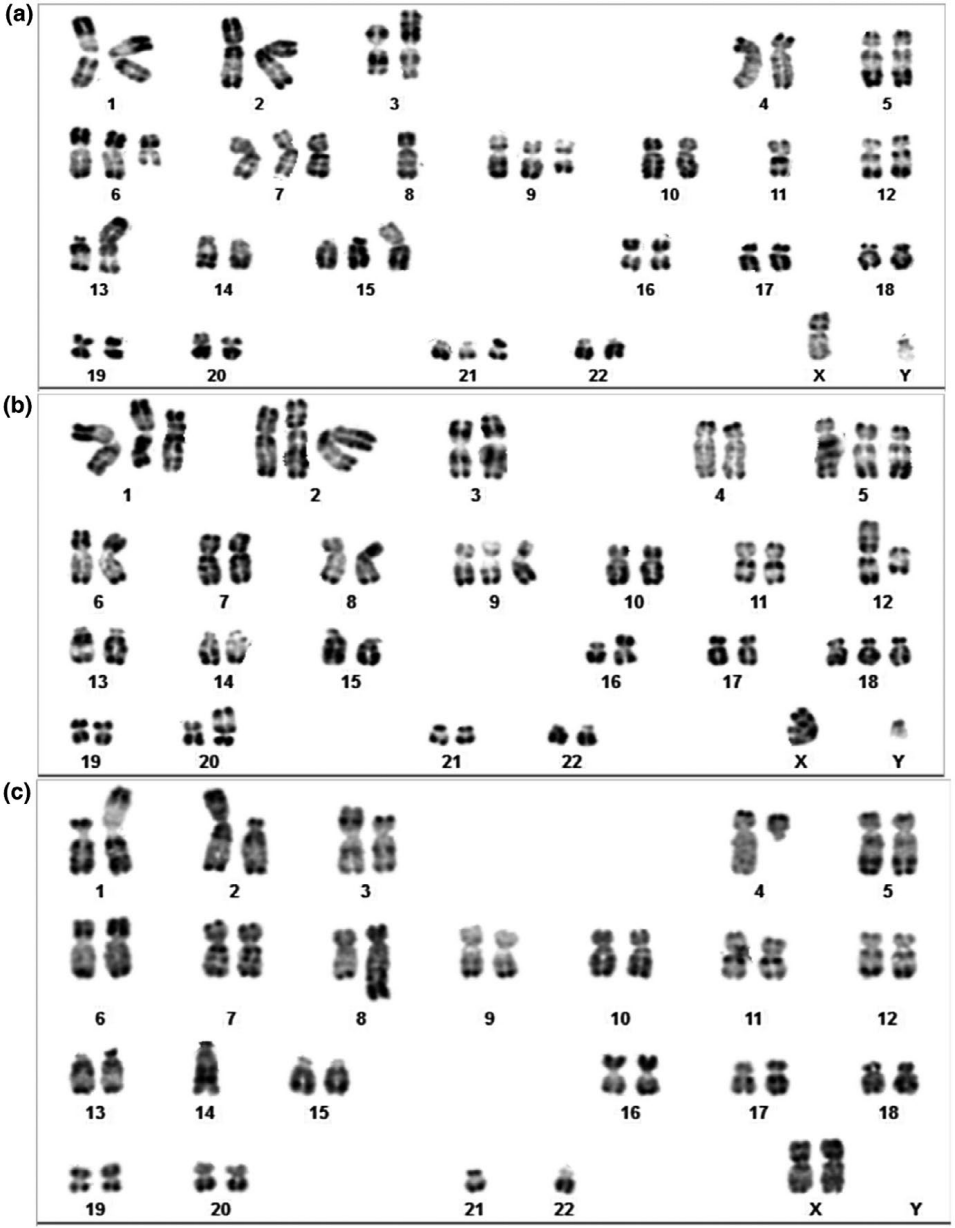
Abnormal bone marrow Karyotypes examples. A, Karyotype of male with 49‐50,XY, der(3)t(3;?)(P22;?),del(3)(p21),+del(6)(q16q23),+7,‐8,+del(9)(21)),‐11,add(13)(p11),+add(15)(P11),+21[cp4]/46,XY[14]; B, karyotype of female with 51,XY,+der(1)(p34p13), +der(2)t(2;?)(p25;?), +5,+9,der(12)t(12;?)(p13;?),del(16)(p12),+18,der(20)t(20;?)(p13;?)[3]/46,XY[22]; C, karyotype of female with 43,XX,der(1)t(1;21)(q11;q11),del(2)(p.11.2p25),t(3;14)(p21;q32), del(4)(q13), t(8;?;1)(q24.2;?;p32),‐14,‐22[19]/46,XX[9]

The rest of the abnormalities are random, nonspecific aberrations to MM such as del(3)(p21)involving *NCKIPSD* (OMIM 606671), del(9)(q21) whose gene is unknown. Two examples of the obtained karyotype results are shown in Figure 3.

Patients in this group were significantly younger than others (median age 54 years old) with male predominance (M/F: 3/1).

##### Nonhyperdiploidy: (Hypodiploid, pseudodiploid, and near‐tetraploid karyotypes)

Myelomas with either hypodiploidy or pseudodiploidy are characterized by various structural chromosomal abnormalities and monosomies.

In our cohort, nonhyperdiploid karyotype (pseudodiploid, hypodiploid or near‐tetraploid) was found in six of 45 cases with abnormal karyotype, representing 13% of the total karyotypes and 60% of abnormal karyotypes. The majority had 43–46 chromosomes with the exception of one case showed a near‐tetraploid (82–87 chromosomes). The most common monosomies are loss of chromosomes: X (*n* = 3), 13 (*n* = 3), 14 (*n* = 1), 16 (*n* = 2), 22 (*n* = 3).

It was usually associated with heterogeneous structural chromosomal aberrations such as t(11;14)(q13;q32) translocation involving *IGH/CCND1* (OMIM 146910)/(OMIM 168461) in four cases, *CMYC* translocation in one case, dup(1)(q21) involving *CKS1B* (OMIM 116900) in one case, IGH translocation (OMIM 146910) in one case, and 13q deletion involving *DLEU1* (OMIM 605765) in one case. An example of the obtained karyotype results is shown in Figure 3. The median age of this group of patients was 57 years old with female predominance (M/F: 2/4). The detailed karyotype results are summarized in Table 2.

### 3.3 FISH results

FISH had demonstrated the presence of chromosomal anomalies in 50% (47/93) which are as follows: the translocation t(4;14) was the most frequent abnormality (14%), followed by the duplication of the long arm of chromosome 1(dup(1q21)) (13%). Next, both the deletion of the short arm of chromosome 17 (del(17p)) (*Cytogenetic location* of *P53* gene) (OMIM 19117)and the *IGH* rearrangement (OMIM 146910) involving other partner chromosomes (12%) then the deletion of the short arm of chromosome 1 (4%). The remaining cases had deletion of 14q32 (*IGH*; 6%) and polyploidy (3%).

#### 3.3.1 IGH translocations

Chromosomal translocations are observed in 50%−70% of the patients with myeloma and over 90% of these translocations involve chromosome 14, which includes the *IGH* locus at 14q32. Most recurrent translocations such as t(4;14) involving *IGH‐MMSET/FGFR3*(OMIM 146910)/(OMIM 134934),t(6;14) involving *IGH‐CCND3*(OMIM 146910)/(OMIM 123834), t(11;14) involving *IGH‐CCND1*, t(14;16) involving *IGH‐MAF*(OMIM 146910)/(OMIM 177075), and t(14;20) involving *IGH‐MAFB*(OMIM 146910)/(OMIM 608968) are regarded as primary cytogenetic events that initiate tumor development.

##### Patients with t(4;14)(p16;q32)(IGH/FGFR3)

**Figure 4:**
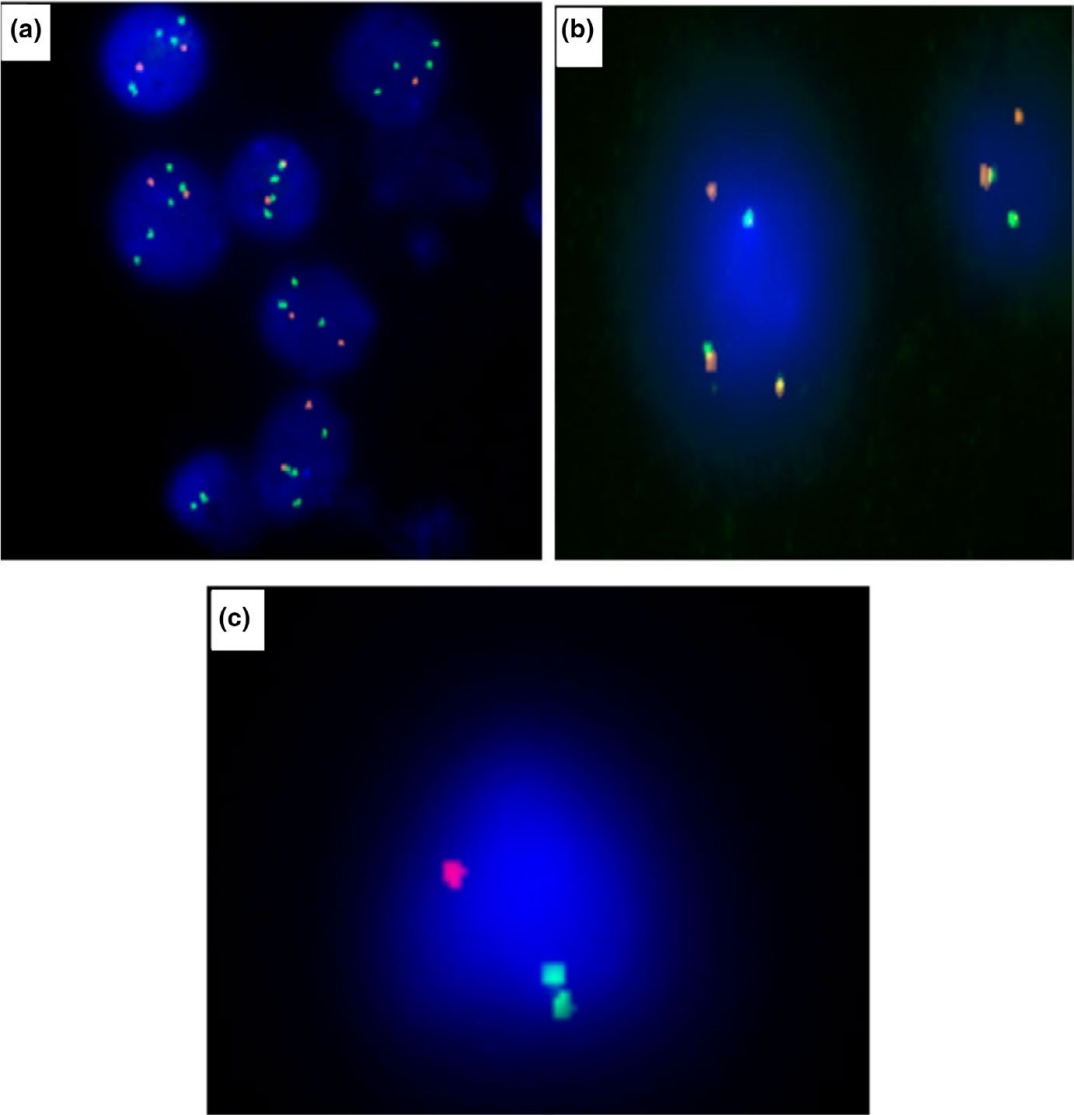
The iFISH technique for multiple myeloma showing different abnormalities. A, 1q amplification (several green signals); B, t(4:14): 1 red, 1 green and 2 fusion signals; C, deletion 17p (one red signal)

By using iFISH analysis, the (4;14) was detected in 13 patients from 93 cases, two patients had a complex karyotype, representing 14% of the total cases and 28% of abnormal FISH. t(4;14) was sole in six cases, associated with one other abnormality in two cases and with two or more abnormalities in three cases. An example of the obtained FISH results was shown in Figure 4. In this group, median age was 62 years old with male predominance (M/F: 8/5).

##### Patients with IGH (14q32) translocation involving unknown partner chromosomes

Eleven patients of all cases had various t(V;14) partners or of an undetermined origin, such as: t(6;14)/*IGH‐CCND3*, t(11;14)/*IGH‐CCND1*, t(14;16)/*IGH‐MAF*, and t(14;20)/IGH‐MAFB accounting for 12% of the total cases and 23% of abnormal iFISH.

The *IGH* rearrangement was sole in six cases, associated with one other abnormality in four cases, and with two others abnormalities in one case.

Patients in this category were younger than the precedent groups (median age 55 years old) with male predominance (M/F: 6/5).

#### 3.3.2 Copy number aberrations

##### Patients with 1q21 duplication

Twelve of the total studied cases had the dup(1q21), accounting for 13% of the total, 25% of the abnormal iFISH. It was sole in five cases, associated with one other abnormality in three cases and with two or more abnormalities in four cases. An example of the obtained FISH results is shown in Figure 4. The median age was 63 years with female predominance (M/F: 5/7).

##### Patients with 17p deletion

The del(17p) was detected among 11 patients by iFISH representing 12% of the total cases and 23% of the abnormal iFISH. It was sole in three cases, associated with one other abnormality in four cases, and with two or more abnormalities in four cases. An example of the obtained FISH results is shown in Figure 4. The median age of this group was 64 years old with no gender predominance (M/F: 6/5).

##### Patients with 1p deletion

We identified a deletion of short arm of chromosome 1 (del(1p)) involving *CDKN2C* (OMIM 603369) in four cases (4% of the total, 8% of the abnormal iFISH). It was sole in two cases, associated with one other abnormality in one case and with two other abnormalities in one case. The median age of this group was 58.5 years old with male predominance (M/F: 3/1).

##### Other abnormalities

Isolated monosomy 14 in was observed in six cases (4% of the total, 13% of the abnormal iFISH), it was sole in four cases and combined to the trisomy of chromosome 4 in two cases.

The median age of this group was 60 years old with male predominance (M/F: 4/2).

**Figure 5:**
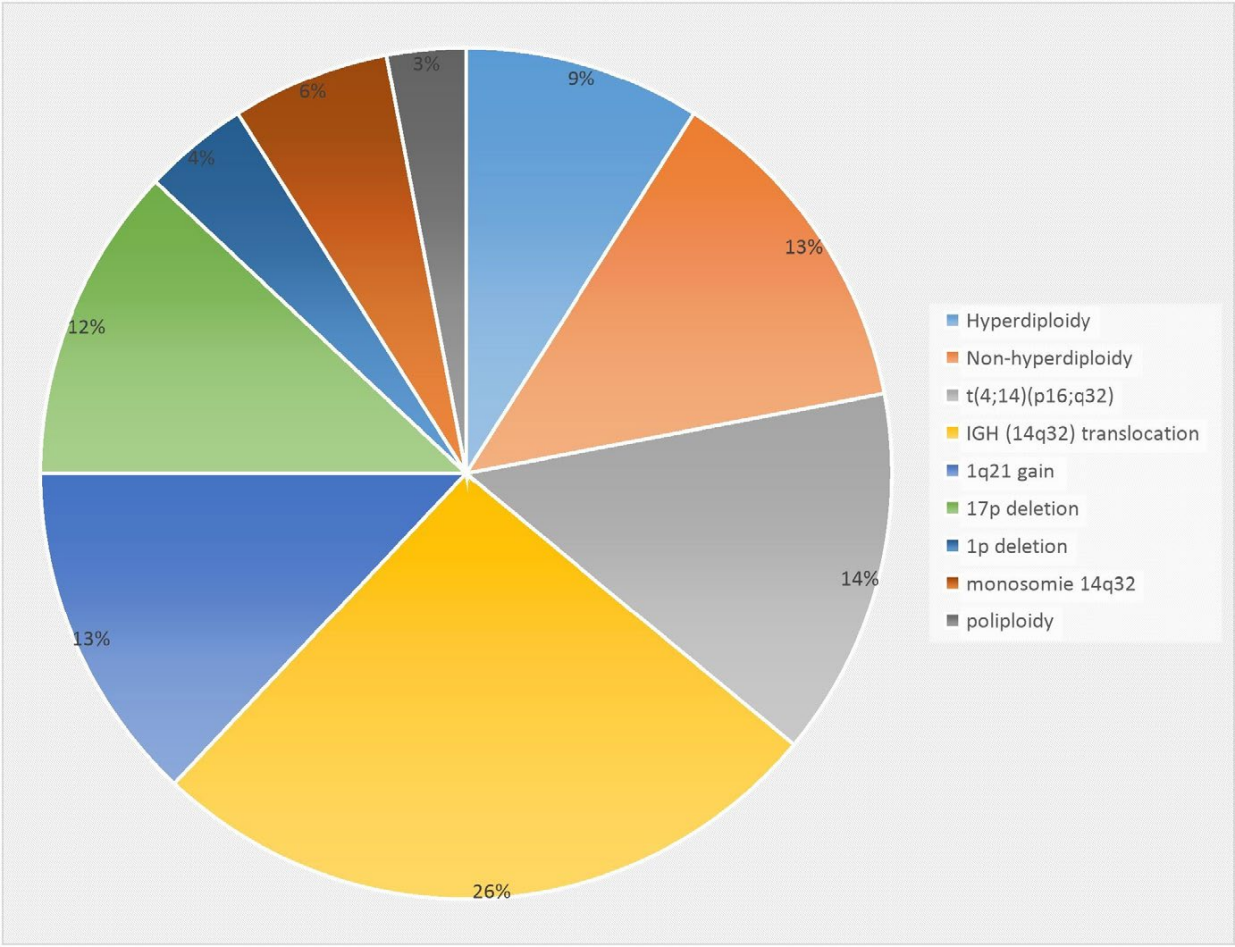
Cytogenetic number and structural abnormalities in 93 cases of multiple myeloma cases

Trisomy of chromosome 17 was seen in one case and polyploidy was highlighted in three cases (Figure 5).

### 3.4 Correlation FISH/karyotype

To show the importance of FISH analysis for MM diagnosis, the karyotype and FISH results were compared for the 45 patients who received both tests. FISH detected the chromosomal abnormalities on the complex karyotype in the 10 patients, for example: the chromosome 1 derivative observed at karyotype was found amplified by FISH as well as the translocation t(11;14) found by karyotype showed a rearrangement of the *IGH* locus by FISH. For the 35 MM patients with normal karyotype, FISH revealed chromosome abnormalities in 16 patients (46%).

## 4 DISCUSSION

Metaphase chromosome analysis in MM can provide the advantage of whole genome analysis, thus it gives a broad image of chromosome aberrations in proliferating plasma cells. However, the low mitotic index of plasma cells makes conventional cytogenetic difficult (Avet‐Loiseauet al., 2007; Mohamed et al., 2007; Saxe, Seo, & Bergeron, 2019), and therefore Chromosomal analysis of clonal PCs in MM is a difficult challenge. On the other hand, iFISH, has greatly increased the detection rate of genetic abnormalities in MM. However, FISH provides limited information (Kishimoto et al., 2016; Mohamed et al., 2007) and detect specific target arrangements as well as chromosomal number changes. To our best knowledge, no cytogenetic study has been published among Moroccan MM patients. Our study aimed to highlight the cytogenetic features of MM in Moroccan patients and compare our results with those from other populations. The Bone marrow of 93 MM patients were included in the current study and analyzed in the National Reference Laboratory between May 2017 and June 2018. The mean age of all patients was 62 years old (range, 30–86 years), which is in coherence with that reported in a other studies (Avet‐Loiseau et al., 2013; Cremer et al., 2005; Kyle et al., 2003; Palumbo & Anderson, 2011).

MM cells are characterized by high genetic instability, resulting in a complex set of numerical and structural chromosomal abnormalities (Lloveras et al., 2004). In newly diagnosed symptomatic patients, the modal chromosome number is usually either hyperdiploid with multiple trisomies or nonhyperdiploid (Mohamed et al., 2007; Saxe et al., 2019). The structural abnormalities include translocations and copy number aberrations such as gains and deletions (Saxe et al., 2019).

Among the 45 cytogenetic analyses, 10 patients (22%) displayed chromosome abnormalities and according to their chromosome number, two groups were identified: a hyperdiploid group with chromosome number greater than 46 was seen in 9% of all cases and the second group with a nonhyperdiploid karyotype was seen in 13%. This percentage (22%) was slightly less than that published in the literature (Avet‐Loiseau, Hulen, et al. 2013; Saxe et al., 2019; Weh et al., 1993). When the karyotype is abnormal, it is generally complex, which is in agreement with previously reported studies (Avet‐Loiseau, Durie et al., 2013).

Different studies including that of Mohamed et al., 2007, suggest that hyperdiploidy is the most common abnormality with a prevalence of 64% of all cases (Mohamed et al., 2007). These results disagree with ours, which show a predominance of nonhyperploidy with a frequency of 13%.

Nonhyperdiploidy was the most common cytogenetic abnormality reported in six cases (three cases pseudodiploid, two cases hypodiploid and one case; 60% of the abnormal karyotype) while 40% (4/10) of them were hyperdiploid. The risk stratification in the hypodiploidy is intermediate (Mikhael et al., 2013). In this group, the most common monosomies are loss of chromosomes: X (*n* = 3), 13 (*n* = 3), 14 (*n* = 1), 16 (*n* = 2), 22 (*n* = 3). The most common structural abnormality is the t(11;14) seen in four cases in addition to one case of translocation 14q32 with unknown partner.

The study of Avet‐Loiseau et al., reported similar results, the *IGH* translocations were found in up to 60% of the patients (Smadja et al., 2003), Calasanz et al. reported a frequency of 22% of the rearrangements of *IGH* among the abnormal cases (Calasanz et al., 1997). In other study, 17% of hyperdiploid MM patients had 14q32 translocations and 63% of cases with 14q32 translocations had a nonhyperdiploid karyotypes (Mohamed et al., 2007). In MM, hyperdiploidy is associated with a good outcome but, patients with both hyperdiploidy and other unfavorable abnormalities such as del (17p), t(4;14) and 1q gain have bad prognosis (Saxe et al., 2019). The risk stratification of hyperdiploidy is standard (Mikhael et al., 2013).

In the current study, Hyperdiploidy was detected in 9% of the total karyotypes studied and involved gain of the odd‐numbered chromosomes 3,5,7,9,11,15,19, and 21. All cases were associated to many structural aberrations, with the deletion of the short arm of the chromosome 1 is the most frequent (in two cases) followed by the translocation affecting band 8q24 *CMYC* and other random nonspecific aberrations. Our findings are in agreement with other studies (Calasanz et al., 1997; Mohamed et al., 2007).

The *IGH* was not detected by karyotype in this group, although, two cases have been identified by FISH analysis. Similarly, in the study by Mohamed et al. the *IGH* translocations were less frequent in the hyperdiploid karyotypes than in the nonhyperdiploid karyotypes (Mohamed et al., 2007; Smadja et al., 2003).

In the study of Avet‐Loiseau et al., Chromosomal abnormalities of 14q32 have been observed in 75% of patients with MM (Avet‐Loiseau et al., 2002) and in 18% of MM patients with different partner chromosomes in Lloveras study (Lloveras et al., 2004). Chromosome rearrangement of 14q32.33 has recurrently occurred with variable partner sites in a Japanese study (Nishida et al., 1997). In our cohort, the 14q32 anomaly was found in 26% of all cases by FISH, including 12% with other unknown partners chromosomes other than chromosome 4, and 14% in the translocation t(4;14). Thus, it is the most frequent anomaly with a percentage of 26%.

In our cohort, the t(4;14) was the most frequent abnormality highlighted by FISH followed by 1q21 duplication and del 17p in 14%, 13%, and 12% of the patients respectively. These results were almost identical to those reported by Avet‐Loiseau et al. (2007) and Avet‐Loiseau et al. (2013).

The t(4;14)(p16;q32) translocation was detected in 10%–15% of the patients with myeloma. The breakpoints are located on distal parts of both chromosomes, and the translocation is undetectable by conventional chromosomal analyses, associated with unfavorable prognosis (Avet‐Loiseau et al., 2007; Neben et al., 2013; Saxe et al., 2019; Table 3), the risk stratification is intermediate (Mikhael et al., 2013). It was the most common translocation among structural abnormalities and his incidence in our cohort was in agreement with the frequencies reported in the literature as shown in the Figure 3.

**Table 3:** Comparison of the distribution of the major structural abnormalities in 93 Multiple Myeloma Moroccan Patients, with others from different populations and their prognosis

Cytogenetic abnormalities	Number of cases	Sole	Associated with other abnormalities	% of Abnormal cases/ FISH	% of the total	% in the literature	Age group	Prognostic impact	Mecanisme
Hyperdiploidy Trisomies of odd numbered chromosomes (*1‐*3‐5‐7‐9‐11‐15‐*17‐*19‐21)	4/45	0	4	40%	9%	50 à 60% Neben et al., 2013, Saxe et al.	54	Standard prognosis unless associated with poor prognosis markers	Primary event ^Rajan & Rajkumar,^ ^2015^ ^, Saxe et al.^
t(4;14)(p16;q32)/*IGH‐MMSET/FGFR3*	13	6	5	28%	14%	10−15%Saxe et al.	52	Adverse	Primary event ^Rajan & Rajkumar,^ ^2015^ ^, Saxe et al.^
IGH translocations	11	6	5	23%	12%	50−70%Saxe et al.	55	Neutral/adverse	Primary event ^Rajan & Rajkumar,^ ^2015^ ^, Saxe et al.^
1q21 gain	12	5	7	25%	13%	35−40% Neben et al., 2013, Saxe et al.	63	Adverse	Secondary event ^Rajan & Rajkumar,^ ^2015^ ^, Saxe et al.^
17p deletion	11	3	8	23%	12%	10% Neben et al., 2013, Saxe et al.	64	Adverse	Secondary event ^Rajan & Rajkumar,^ ^2015^ ^, Saxe et al.^
1p deletion	4	2	2	8%	4%	30% Neben et al., 2013, Saxe et al	58	Adverse	Secondary event
Poliploidy	3	3	—	6%	3%	50% Saxe et al	67	Favorable	Primary event ^Rajan & Rajkumar,^ ^2015^ ^, Saxe et al.^
Combined or Isolated monosomy 14	6	4	2	13%	6%	No data available	60	Adverse	Primary event ^Rajan & Rajkumar,^ ^2015^ ^, Saxe et al.^

Abbreviations: FISH, fluorescence in situ hybridization; IgH, immunoglobulin heavy chain.

1q21 gain is the most frequent structural abnormality, observed in 35%–40% of the patients with MM (Saxe et al., 2019). 1q gain is an independent poor prognostic factor (Neben et al., 2013; Saxe et al., 2019; Table 3). The frequency of this abnormality in our population was significantly less than that reported in one study published in the literature (Figure 3).

Chromosome 17p deletion is considered as a secondary event. It is observed in around 10% of patients with newly diagnosed with MM (Avet‐Loiseau et al., 2007; Neben et al., 2013; Saxe et al., 2019). *TP53* deletion in myeloma is a high‐risk marker associated with adverse prognosis (Saxe et al., 2019; Table 3). The incidence of this abnormality was significantly in concordance with the literature as shown in Figure 3.

The most important recurrent genetic abnormalities present in the malignant plasma cells that displayed a strong prognostic power are the t(4;14), del(17p) (Avet‐Loiseau et al., 2013) and the integration of the chromosome 1 abnormalities(1q21 duplication and 1p32 deletion) into prognostic stratification models is not yet consensual^12^. Thus, 14% of our patient had an intermediate risk, 12% had a high risk.

To summarize our finding, Interphase FISH was able to detect genomic abnormalities in almost 46% of the patients with normal Karyotype, about two times more frequently than conventional chromosomal banding (22%). It had demonstrated 50% of chromosomal abnormalities, this frequency was low than that reported in other studies (Avet‐Loiseau et al., 2007; Kishimoto et al., 2016; Wang, Wu, & Yang, 2018).

## 5 CONCLUSION

This study was the first of its kind to focus on the cytogenetic profile of multiple myeloma in Moroccan patients. It plays an important role in the classification and prognosis of MM patients, as it makes it possible to stratify patients into prognostic groups as well as the association between emerging therapeutic approaches in MM, based on the results of in situ cytogenetic hybridization and conventional fluorescence (FISH).

The t(4; 14) and del (17p) are very important for the prognosis and the therapy which allow the differentiation between MGUS and MM. These aberrations confer a poor prognosis and have a negative impact on overall survival. Patients with these genomic aberrations should be treated with targeted therapy.

Our results revealed that conventional cytogenetics remains an important tool for elucidating complex and diverse genetic abnormalities in MM. Although iFISH is more sensitive than the classic karyotype, the combination of the two analyzes can increase the rate of detection of abnormalities.

Due to the heterogeneity of myeloma abnormalities and the high level of mutations, we recommend new molecular biology tools as complementary techniques such as gene expression profiling (GEP; a routine technique, very useful for the patient, since it makes it possible to know which genes it overexpresses and therefore to identify potential therapeutic targets) and/or the SNP array/CGH array (a sensitive, reliable technique applicable on an individual level).

## CONFLICT OF INTEREST

The authors declare that there are no conflicts of interest.

## AUTHOR CONTRIBUTION

Hasna Hamdaoui carried out the cytogenetic study, interpreted the data and drafted the manuscript. Oumaima Benlarroubia carried out the cytogenetic study. Oum Kaltoum Ait boujmia revised and edited the first draft of the manuscript. Hossein Mossafa supervised the work and interpreted the data. Karim ouldim analysed the data. Aziza Belkhayat analysed the data and reviewed the manuscript. Imane Smyej and Houda Benrahma read the paper. Hind Dehbi revised and edited the manuscript. Fatima Chegdani involved in provision of research ideas, reviewed and edited the paper before submission.

## Data Availability

The prospective data that support the findings of this study are available from the corresponding author upon reasonable request.
